# Human Polyomavirus BK Genome Analysis in BKPyV Induced Rodent Cell Lines

**DOI:** 10.1002/mbo3.70061

**Published:** 2025-09-11

**Authors:** Setsuko Shioda, Fumio Kasai, Midori Ozawa, Azusa Ohtani, Masashi Iemura, Ken Watanabe, Arihiro Kohara

**Affiliations:** ^1^ National Institute of Biomedical Innovation Health and Nutrition Ibaraki Osaka Japan; ^2^ Cell Engineering Division BioResource Research Center Tsukuba Japan; ^3^ Center for Stem Cell and Regenerative Medicine Tokyo Medical and Dental University (TMDU) Tokyo Japan

**Keywords:** BK polyomavirus, In‐1024, noncoding region, plaque morphology mutant 522, Vn1919, Vn‐324, wild‐type 501

## Abstract

In this study, we analyzed the BK polyomavirus (BKPyV) genome derived from three rodent cell lines established from experimentally induced tumors by injecting BKPyV into newborn rodents. Three cell lines (Vn‐324, In‐1024, and Vn1919) were recently deposited in the JCRB Cell Bank (Japanese Collection of Research Bioresource Cell Bank). Vn‐324 was established from a hamster choroid plexus papilloma induced by BKPyV Gardner strain wild‐type 501 (*wt‐501*). This cell line was reported to be negative for the large T‐antigen using indirect immunofluorescence. In this study, we examined the large T‐antigen expression using the reverse‐transcriptase‐polymerase chain reaction (RT‐PCR). In‐1024 cells were established from hamster insulinoma. The strain of BKPyV from which were induced has not been reported. Vn1919 was established from a mouse ependymoma induced by the plaque morphology mutant 522 (*pm‐522*). The noncoding control region (NCCR) of BKPyV derived from Vn‐324 genomic DNA and *wt*‐501 had the same structure, whereas the NCCR of BKPyV derived Vn1919 genomic DNA and *pm‐522* had the same structure. But the NCCR derived In‐1024 was unique. We revealed that BKPyV derived from In‐1024 genomic DNA had a large deletion in the viral proteins 1, 2, and 3 (VP1,(VP1, VP2, and VP3) coding region. This variant may be a proliferation‐defective mutant, which was expanded in human embryonic kidney cells with other mutants. These findings provide insights into the role of NCCR mutations in viral oncogenesis.

## Graphical abstract

In this study, we analyzed the BK polyomavirus (BKPyV) genome derived from three rodent cell lines established from BKPyV induced tumors. BKPyV derived one cell line had a large deletion. The noncoding control region (NCCR) of BKPyV derived from these cell lines had the same structure as viruses induced tumors.

**Abbreviations**
BKPyV: BK polyomavirus
HBSS: hanks’ balanced salt solution
LT‐ag: large T‐antigen
MCPyV: Merkel cell polyomavirus
NCCR: non‐cording control region
PBS: phosphate‐buffered saline
pm‐522: plaque morphology mutant‐522
RT‐PCR: reverse transcriptase‐polymerase chain reaction
SV40: simian virus 40
Tfrc: transferrin receptor gene
VP1: viral protein 1
VP2: viral protein 2
VP3: viral protein 3
wt‐501: wild‐type‐501

## 1 Introduction

**Figure 1:**
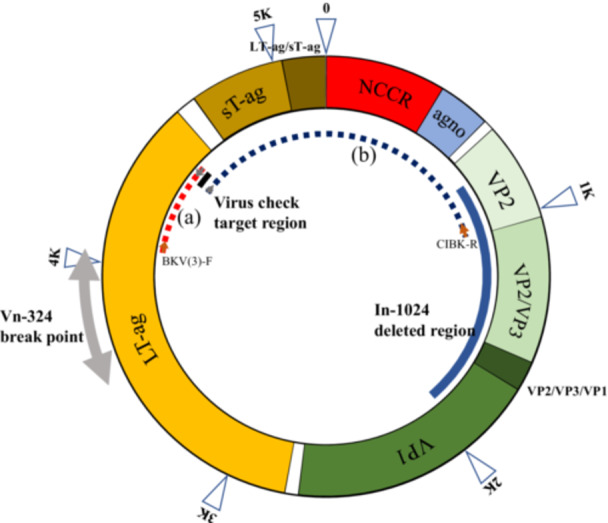
The complete genome of BK polyomavirus strain Gardner, which is 5190 bp in length and includes the NCCR and coding regions for agnoprotein, VP1, VP2, VP3, LT‐ag, and sT‐ag. The black square indicates the target region for virus check, while the long blue square represents the deleted region of BKPyV derived from In‐1024. The gray area indicates the region containing the integration breakpoint of BKPyV derived Vn‐324 into the host genome. Fragment (a) is the PCR product of primer set BKV(3)‐F and BK JC‐BK‐R for the LT‐ag expression assay, and fragment (b) is the PCR product BK JC‐F and CIBK‐R for NCCR structure analysis.

BK polyomavirus (BKPyV) is a small, non‐enveloped virus with a circular double‐stranded DNA genome of approximately 5 kb, belonging to the Polyomavirus family. The name of this virus is derived from the patient's initials, from whom it was first isolated (Gardner et al. 1971; Calvignac‐Spencer et al. 2016; DeCaprio and Garcea 2013). It is known to infect approximately 80% of the human population during early childhood (Rinaldo et al. 2013; Egli et al. 2009; Kean et al. 2009). Following the primary infection, BKPyV persists latently in the urinary tract. Asymptomatic shedding of BKPyV into the urine of immunocompetent individuals has been observed in several patients, and nephritis has sometimes been reported (Fioriti et al. 2005; Kenan et al. 2015). The BKPyV genome contains the noncoding control region (NCCR), which is approximately 400 base pairs (bp) long and is located between the early and late regions (Figure 1). The NCCR of BKPyV in healthy immunocompetent individuals and the NCCR derived from immunocompromised kidney transplant patients in the early stages of BKPyV‐associated nephropathy are archetypes. However, when immunosuppression is not readily reduced to permit specific T cells to resume control over BKPyV replication, viral variants with rearranged NCCRs emerge and are associated with higher plasma viral loads and more severe renal allograft pathologies. However, the molecular mechanisms leading to NCCR rearrangements in DNA viruses at such higher frequencies are intriguing and unclear (Bethge et al. 2015; Gosert et al. 2008). A previous study reported that BKPyV NCCR rearrangement could lead to oncogenic transformation in urothelial cancer in immunosuppressed patients (Müller et al. 2018). The oncogenicity of BKPyV in humans has long been debated. Uchida et al. reported that the BKPyV samples originating from a single stock of Gardner's original strain were poly‐oncogenic in hamsters and that insulinoma inducibility differed markedly among the virus samples (Uchida et al. 1979). Watanabe et al. reported that plaque morphology mutant 522 (*pm‐522)* of BKPyV was a viable deletion mutant capable of inducing insulinomas in hamsters, whereas a cloned *wild‐type 501 (wt‐501)*, induced brain tumors and osteosarcomas, but not insulinomas (Watanabe et al. 1979). Experimentally induced tumors were diagnosed based on symptoms and organ and blood glucose levels, and cell lines were established from these tumors. BKPyV genome sequences were detected in all the cases. The copy number of BKPyV DNA detected in the cell lines ranged from a few to multiple copies, depending on the cell line (Yogo et al. 1980). Some tumors contained nonintegrated free viral DNA in addition to the integrated BKV genome (Yogo et al. 1980). Three cell lines, Vn‐324, Vn1919, and In‐1024, were deposited in the JCRB Cell Bank (Japanese Collection of Research Bioresource Cell Bank). These cell lines were established from BKPyV induced tumors in newborn rodents. Vn‐324 and Vn1919 have been reported to be involved in cell line establishment and characterization (Yogo et al. 1981; Aizawa et al. 2011). However, there have been no reports on In‐1024. We analyzed the BKPyV genome structure derived from two hamster cell lines and one mouse cell line.

The BKPyV that induced rodent tumor cells mutated while growing in human embryonic kidney cells. BKPyV and other polyomavirus JCPyV are latent in most people. When a person becomes immunocompromised for some reason, these viruses reactivate and mutate. In addition, a new highly carcinogenic Merkel Cell Polyomavirus has also been discovered. Clarifying the relationship between BKPyV mutations and carcinogenesis could be an important issue for human health.

## 2 Materials and Methods

### 2.1 Biological Analysis

#### 2.1.1 Cells and Culture Conditions

Three cell lines, Vn‐324, Vn1919, and In‐1024, were cultured in Dulbecco's Modified Eagle's medium (Gibco, Thermo Fisher Scientific) with 10% fetal bovine serum.

Vn‐324 (JCRB1742) is a polygonal epithelial cell line that was established from a choroid plexus papilloma induced by the purified variant *wt‐501* Gardner's strain of BKPyV in a Syrian golden hamster and was confirmed to be large T‐antigen (LT‐ag), negative using immunofluorescence (Yogo et al. 1981). Vn1919 (JCRB1361) is an ependymoma cell line co‐expressing neuroglial and neuronal characteristics and was established from a mouse ependymoma induced by BKPyV purified variant *pm‐522* in BALB/c mice (Aizawa et al. 2011). The virus inoculated into hamsters or mice was grown in human embryonic kidney cells, collected using cytopathic effect (CPE) as an indicator. The highly concentrated virus solution was collected as a band by cesium chloride density gradient ultracentrifugation. Finaly high concentrated virus in PBS was inoculated into the lateral ventricle of hamsters or mice within 24 h after birth to conduct carcinogenesis experiments (Uchida et al. 1979).

#### 2.1.2 Immunofluorescence Staining

Immunofluorescence staining was performed using the standard methods (Fukawatase et al. 2014). These three cell lines were cultured in 35 mm^2^ dishes of cell culture grade in growth medium. The cells were then fixed with 4% paraformaldehyde for 10 min after being non‐shaking washed with Hanks' Balanced Salt solution (HBSS). After treatment with 0.2% Triton‐X in phosphate‐buffered saline (PBS) for 10 min at room temperature (20°C–25°C), the cells were washed with HBSS and then treated with 5% normal goat serum in 1% bovine serum albumin in PBS to block nonspecific staining. The cells were then incubated for 1 h at room temperature (20°C–25°C) with anti‐simian virus 40 (SV40) T‐antigen Ab (PA b 416: ab16879, Abcam) diluted at a ratio of 1:50 in 1% bovine serum albumin‐PBS. Next, the cells were non‐shaking washed three times with HBSS and incubated with the secondary antibody along with 4',6‐diamidino‐2‐phenylindole for 30 min at room temperature. After washing with HBSS, the cells were mounted using a fluorescent mounting medium (DAKO cat# 3023) (Kiprianova et al. 2019) and covered with a cover glass. Images were captured using a fluorescence microscope (All‐in‐one microscope BZ‐X810, Keyence Japan).

### 2.2 Molecular Analysis

#### 2.2.1 DNA and RNA Extraction

Genomic DNA and total RNA were extracted from each cell line using the AllPrep DNA/RNA Mini Kit (QIAGEN, Tokyo, Japan). To eliminate DNA contamination from the isolated total RNA, RNase free‐DNase I (QIAGEN) was used to digest the extracted RNA at room temperature for 15 min, following the manufacturer's protocol.

#### 2.2.2 LT‐ag Expression Analysis Using Reverse Transcriptase‐Polymerase Chain Reaction (RT‐PCR)

The expression of LT‐ag in the three cell lines was analyzed using RT‐PCR after cDNA synthesis using a High‐Capacity cDNA Reverse Transcription Kit (Applied Biosystems) following the manufacturer's protocol. Total RNA was extracted from all three cell lines. PCR was performed using the primers BKV(3)‐F and BK JC‐BK‐R. The PCR product was located within the LT‐ag coding region near the 3énd position (Figure 1. fragment [a]). The sequence of all primers used in this paper and their locations on the reference genes, BK polyomavirus strain Gardner (GenBank: LC029411), are shown in Table 1. AS for the primers for virus testing (BK JC‐F, BK JC‐BK‐R), these three cell lines are positive in real‐time PCR of genomic DNA. For primer BKV(3)‐F, there is a site for Vn1919, which has almost no deletion in the detected BKPyV virus genome. It is not included in the deletion site even for In‐1024, which has a large deletion. Also, for Vn‐324, detected BKPyV genome is integrated into host genome in the coding region of the LT‐ag, the primer site is outside the breaking point.

**Table 1:** Primer sequences and location on reference genes, BK polyomavirus strain Gardner. Reference genome is BK polyomavirus DNA, complete genome, strain Gardner (Accession: LC029411).

Primer name	Sequences	Location (BKV Gardner strain)
**CIBKV‐R**	AGCCATGCCTGATTGCTGATAGAG	1023–1000
**BK JC‐F (virus check)**	GGAAAGTCTTTAGGGTCTTCTACCTTT	4425–4451
**BK JC‐BK‐R (virus check)**	GATGAAGATTTATTTTGCCATGAAG	4543–4519
**BKV(3)‐F**	CACCTGCTTTGTTTCTTCAGGC	4037–4058

Amplification was performed using KOD FX DNA Polymerase (TOYOBO, Japan), following the manufacturer's protocol. The resulting products were then electrophoresed on 1.2% agarose gels. (E‐Gel Agarose Gels with SYBR Safe, E‐Gel PowerBase, Invitrogen), and DNA bands were visualized and photographed using Gel Doc XR+ with Image Lab Software (BIO‐RAD).

#### 2.2.3 BKpyV Detection and Copy Number Analysis Using Digital PCR

BKPyV DNA detection was performed using the TaqMan probe method for real‐time PCR, as previously described, with a primer probe set for virus detection (Shioda et al. 2018).

A QuantStudio 3D Digital PCR System (Thermo Fisher Scientific) was used to examine the copy number of BKPyV detected in the genomic DNA of the three cell lines as previously described (Shioda et al. 2017). Briefly, the PCR reaction mixture was loaded onto digital PCR 20 K chips, and the PCR was performed using primers and probe for virus checking. The genomic DNA of the cell lines was diluted so that the ratio of positive and negative results for the 20 K chip was appropriate. The copy numbers per µg DNA were calculated from the copy number/µl detected as a highly reliable value (The error between three or four measurements of the same sample is less than 10%.). TaqMan Copy Number Reference Assay for the mouse transferrin receptor gene (Tfrc) served as the standard reference for copy number analysis (Applied Biosystems) of the mouse cell line Vn1919 and cloned cell lines. As for the hamster cell line, it was reported that a single copy of the BKPyV genome was integrated into the Vn‐324 genome DNA (Yogo et al. 1981). The copy numbers of In‐1024 were compared with those of Vn‐324 (Table 2).

**Table 2:** Copy number of BKPyV DNA sequences per genome, assayed with Quant Studio 3D Digital PCR. (a) Mouse cell line and clone cells and (b) Hamster cell lines. Copy number reference for mouse cell lines is TaqMan Copy Number Reference Assay for mice (Tfrc). The copy numbers per µg genomic DNA shown in the table were calculated from reliable values within 10% error of measurements from the same sample.

(a) Mouse cell line and clone cells				
Cell no.	Cell name	BKV (copies/µg DNA)	Mouse copy number reference (copies/µg DNA)	BKV (copies/genome)
JCRB1361	Vn1919	9.70E + 04	2.90E + 05	0.7
JCRB1365	Vn1919cl14	1.30E + 05	2.00E + 05	1.3
JCRB1367	Vn1919cl49	1.62E + 05	2.98E + 05	1.1
JCRB1368	Vn1919cl59	1.28E + 05	2.23E + 05	1.1

(b) Hamster cell lines			
Cell no.	Cell name	BKV (copies/µg DNA)	BKV (copies/genome)
JCRB1742	Vn‐324	0.15E + 05	1
JCRB1737	In‐1024	2.12E + 05	14.1

#### 2.2.4 Viral Genome Analysis

The levels of the BKPyV derived from the three rodent tumor cell lines induced with BKPyV Gardner's strain variants were analyzed. The primers used in this study were designed using GENETYX gene analysis software. The reference sequences used were BK Polyomavirus DNA complete genome of strain Gardner (GenBank: LC029411). Additionally, primers for viral checks designed to control the quality of cell lines (Shioda et al. 2018) were also used. PCR was performed using KOD FX DNA Polymerase (TOYOBO, Japan), following the manufacturer's protocol. The resulting products were electrophoresed on a 1.2% agarose gel (E‐Gel Agarose Gels with SYBR Safe, E‐Gel PowerBase, Invitrogen), and the DNA bands were visualized and photographed with Gel Doc XR+ using Image Lab Software (BIO‐RAD). A single band of the PCR product was purified using QIAquick Spin Columns (QIAGEN, Germany) and analyzed by standard Sanger sequencing. The PCR product of BK JC‐F and CIBKV‐R was used to identify NCCR sequences in the BKPyV derived from Vn‐324 and Vn1919 (Figure 1, fragment [b]). For genome structure analysis, we obtained the whole‐genome sequences of BKPyV derived from In‐1024 using primer walking (Supporting information Data 1), because the PCR products of BK JC‐F and CIBKV‐R were not obtained from the In‐1024 genomic DNA. To compare the NCCR structure with variants derived from these three cell lines, we analyzed the database sequences of Human papovavirus BK, Gardner's original strain, wild type 504, early transcription control region (putative) (K02014.1), as well as Human papovavirus BK, Gardner's original strain mutant pm‐522, early transcription control region (putative) (K02015.1), and the Human BK virus (strain Dunlop) genome (GenBank V01108.1) was analyzed. The BKPyV archetype, BK (WW) was derived from patient urine (Markowitz and Dynan 1988), and the sequence and structure of the NCCR have been reported. The NCCR is divided into five transcription factor‐binding blocks, O, P, Q, R, and S. In this study, we examined NCCR homology in three BKPyV‐derived rodent cell lines and viral variants (Moens et al. 1995; Moens and Rekvig 2001).

## 3 Bioinformatic Analysis

### 3.1 Viral Whole Genome Sequencing With Next‐Generation Sequencing

The BKPyV genomes in In‐1024, Vn‐324, and Vn1919 were examined by target sequencing using an AmpliSeq custom panel designed by the AmpliSeq designer (Thermo Fisher Scientific). The panel was based on the complete sequence data of the Gardner strain of BK polyomavirus (ACCESSION: LC029411) as a reference and consisted of 27 amplicons covering the entire 5190 bp genome (Supporting information Data 2). Sequence libraries and templates were prepared using the Ion AmpliSeq Kit for Chef DL8 (Thermo Fisher Scientific, A29024) and the Ion PGM Hi‐Q View Chef Kit (Thermo Fisher Scientific, A29902), respectively. Sequencing was run on the Ion PGM using the Ion PGM Hi‐Q View Sequencing Kit (A30044) and the Ion 318 Chip v2 (Thermo Fisher Scientific, 4488150). Reads were aligned to the Gardner strain reference and analyzed using the Ion Torrent Suite (Thermo Fisher Scientific) and the Integrative Genomics Viewer.

## 4 Results

### 4.1 BKV LT‐ag Detection

The genome position 4425–4543 (Figure 1) in the LT‐ag coding region was used as the target region for the virus check test to ensure quality control of cell lines for BKPyV cell lines (Shioda et al. 2018). Target DNA was detected in the genomic DNA of three cell lines, In‐1024, Vn‐324, and Vn1919, using real‐time PCR (Supporting information Data 3).

**Figure 2:**
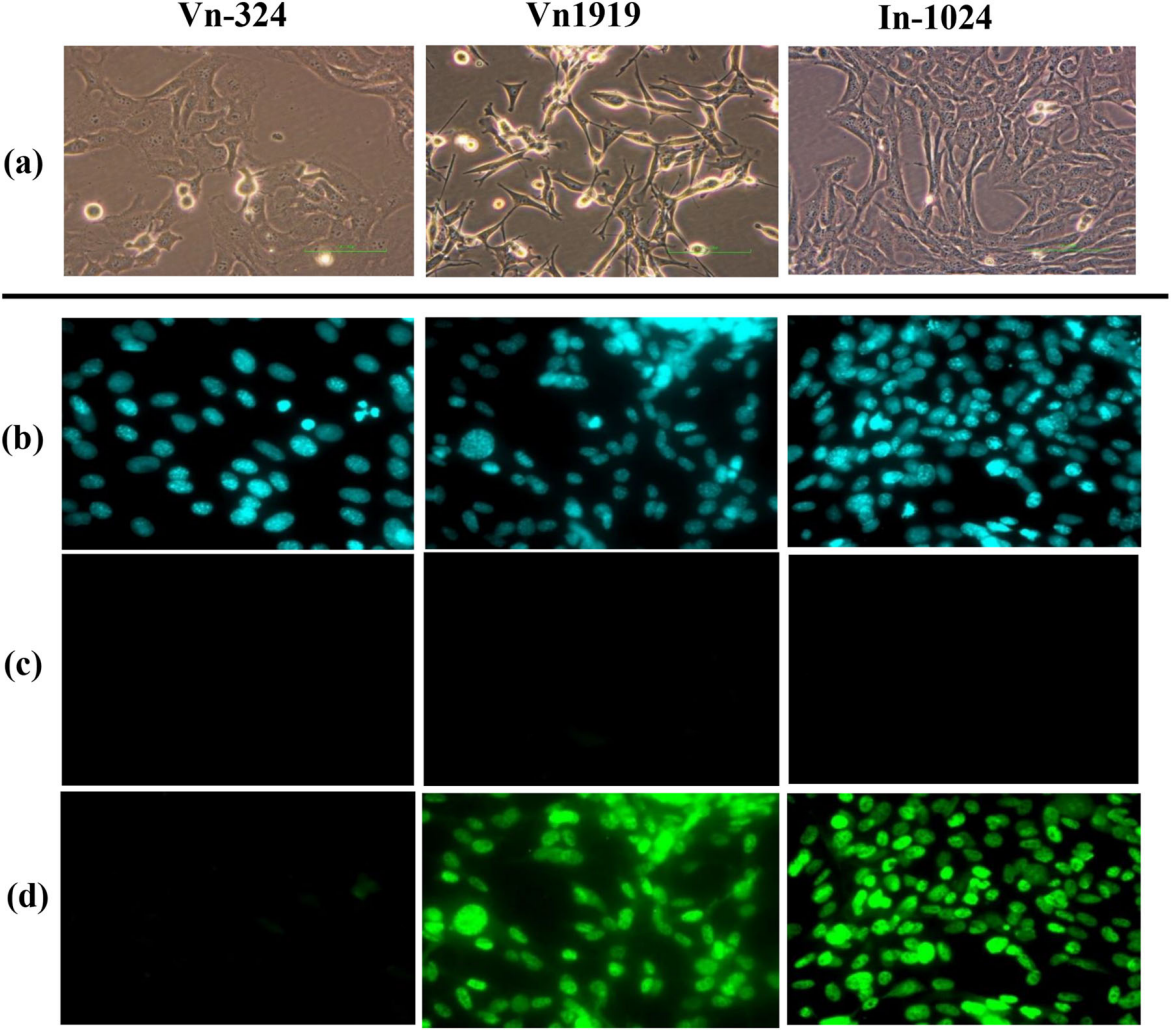
The morphology in the light microscope and immunofluorescence staining LT‐ag of three cell lines. (a) Phase‐contrast image magnification of 200 for three cell lines. Vn‐324 is a polygonal epithelial cell line established from choroid plexus papilloma induced with BKPyV *wt‐501* in Syrian golden hamster. Vn1919 is an ependymoma cell line co‐expressing neuroglial and neuronal character, established from mouse ependymoma induced with BKPyV mutant *pm‐522* in BALB/c mouse. In‐1024 is a polygonal cell line established from BKPyV‐induced insulinoma in Syrian golden hamster. Scale bars represent 100 µm. (b) DNA was stained with DAPI (blue), 600 magnification. (c) Isotype control of immunofluorescent staining, 600 magnification. (d) Anti‐SV40 LT‐ag mouse monoclonal antibody and mouse IgG Alexa488 staining, 600 magnification. At Vn1919 and In‐1024, the nuclei of all cells were stained, but at Vn‐324, none of the cells were stained.

Immunofluorescence staining was used to detect BKPyV LT‐ag in In‐1024 and Vn1919 cells but not in Vn‐324 cells (Figure 2).

### 4.2 LT‐ag Expression

**Figure 3:**
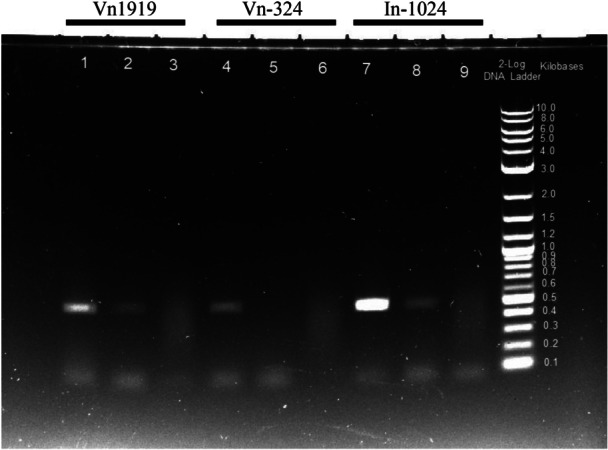
Analysis of LT‐ag expression using RT‐PCR. LT‐ag PCR product is detected in Vn1919 and In‐1024 but not in Vn‐324. Template: genomic DNA (lanes 1, 4, and 7), cDNA (lanes 2, 5, and 8), RNA (lanes 3, 6, and 9) Marker: 2‐Log‐Ladder (NEW ENGLAND BioLabs) Primer: BKV(3)‐F and BK JC‐R (large T‐ag 3'‐end), expected band size: 507 bp. KOD FX (TOYOBO) 3 step PCR (Pre denature 94°C 2 min, Denature 98°C 15 s, Anealing 60°C 30 s, Extention 68°C 1 min, 35 cycles). Lane and Band Analysis of Image Lab (BIO‐RAD), volume of intensities within the band boundaries (lane 1: 37,201,792 lane 2: 27,355,072 lane 4: 29,432,256 lane 7: 50,618,624 lane 8: 25,928,576).

We analyzed LT‐ag expression in three cell lines, Vn1919, Vn‐324, and In‐1024, using RT‐PCR. The target region for PCR was located near the 3énd of LT‐ag, and the expected product size was 507 bp (Figure 1, fragment [a]). The expected band size was detected in the genomic DNA of all three cell lines (Figure 3, lanes 1, 4, and 7).

Following cDNA synthesis, bands of the same size were detected for Vn1919 (lane 2) and In‐1024 (lane 8). However, we did not detect any PCR products for Vn‐324 (lane 5). Therefore, we concluded that LT‐ag was not expressed in Vn‐324.

### 4.3 Copy Number of BKPyV DNA Sequences Per Genome

The copy number of BKPyV per genome in each cell line was calculated using digital PCR (Table 2). Vn1919 had one copy per genome when compared with the TaqMan Copy Number Reference Assay for mice (Tfrc). In contrast, the In‐1024 genomic DNA was compared with the hamster cell line Vn‐324, which was reported to contain one copy of integrated BKPyV in its genomic DNA (Yogo et al. 1981). The results showed that approximately 14 copies of BKPyV DNA per genome were detected in the In‐1024 genomic DNA.

### 4.4 Episomal Virus DNA Detection

**Figure 4:**
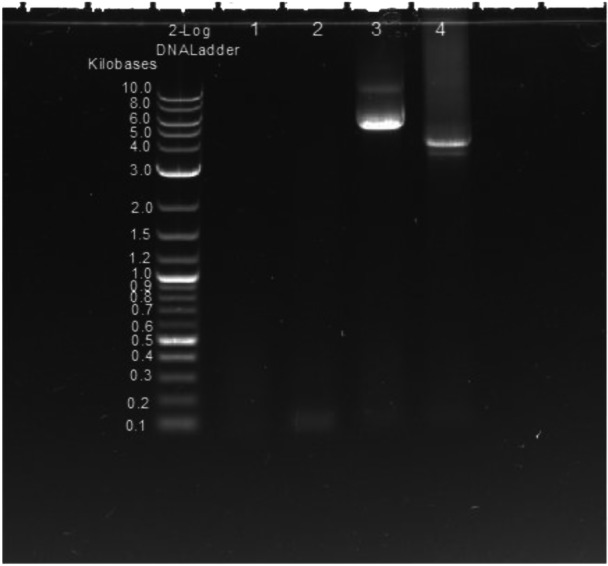
Long PCR products with BKV virus check primers. PCR products with primer set (BK JC‐F and BK JC‐BK‐R) for virus check using genomic DNA of cell lines as a template. PCR protocol was changed to a long extension time for 5 min (Pre denature 74°C 2 min, Denature 98°C 15 s, Annealing 60°C 30 s, Extension 68°C 5 min). No PCR product was detected from spontaneously established Syrian hamster fetus fibroblast transformed cell line DSPT200 (JCRB0217) (lane 1), which was used as a negative control. Lane 2 is Vn‐324, but the detected viral genome is integrated into the host genome, so the PCR product size was 119 bp. Lane 3 is Vn1919. If the detected viral genome is linear, the PCR product size is 119 bp, but a band of almost the entire genome size of BKPyV was detected. The viral genome may exist as a circular episome. Lane 4 is In‐1024. A similarly large band was detected. The detected viral genome had about 1 Kb deletion, so it is almost the size of the entire genome, existing as a circular episome.

Although the PCR product size of the primer set for the virus check (BK JV‐F and BK JC‐BK‐R) was expected to be 119 bp, long extension time (5 min) PCR using Vn1919 and In‐1024 genomic DNA as templates resulted in the appearance of large size products, approximately 4–5 kb, which is close to the whole‐genome size (Figure 4, lanes 3 and 4). However, when Vn‐324 genomic DNA was used as a template, only a 119 bp band was detected (Figure 4, lane 2). A 5 kb band was detected in Vn1919 (lane 3), and a band under 4 kb was detected in In‐1024 (lane 4).

### 4.5 Sequence Analysis

**Figure 5:**
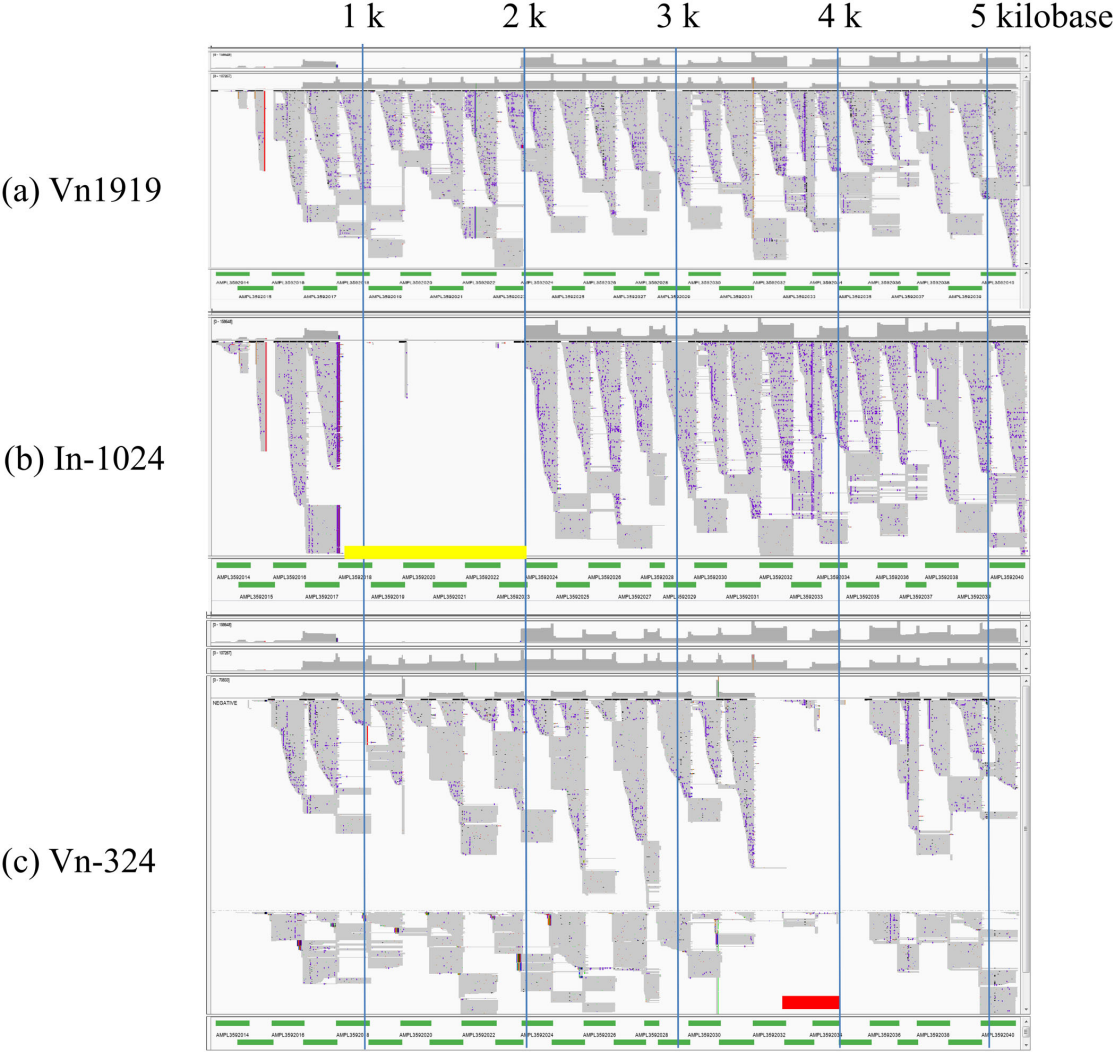
BKPyV genome analysis using NGS mapped to the BKPyV Gardner reference strain. The BKPyV genome derived from In‐1024, Vn‐324, and Vn1919 was examined by target sequencing using an AmpliSeq custom panel developed by the AmpliSeq designer (Thermo Fisher Scientific). The panel was based on the complete sequence data of the Gardner strain of the BK polyomavirus (ACCESSION: LC029411) as a reference and consisted of 27 amplicons (green lines, Supplemental Data 2) covering the entire 5190 bp genome. The deletion regions of the BKPyV derived In‐1024 and Vn‐324 were confirmed. The yellow line indicates the deletion site of In‐1024. The red line indicates the likely region of integration site of Vn‐324. The gaps around the red line where no amplicon was formed are thought to be due to mutations that occurred during integration into the host genome.

The long PCR product derived from In‐1024, with a large band of approximately 4 kb, was sequenced by primer walking. The analysis revealed that the BKPyV genome derived from In‐1024 had a deletion of 1184 bp (from 798 bases to 1981 bases), which is approximately one‐fourth of the size whole viral genomic DNA. This deleted region included VP2, VP3, and approximately half of the VP1 coding region. In contrast, the BKPyV genome detected in Vn1919 did not contain a large deletion. The genome map of BKPyV derived from the three cell lines, confirmed by sequence analysis of the PCR products using next‐generation sequencing is shown in Figure 5.

#### 4.5.1 NCCR Structure Analysis

The PCR products generated by the BK JC‐F and CIBKV‐R primers contained NCCR and agnoprotein sequences totaling approximately 1.5 kb (Figure 1, fragment [b]). We analyzed the NCCR sequences of BKPyV derived from Vn‐324, Vn1919, and In‐1024 for comparison with the virus variants.

**Figure 6:**
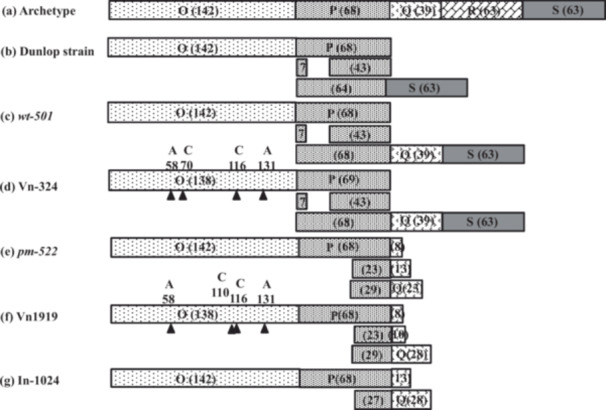
The NCCR structure of BKPyV derived from three rodent cell lines and the archetype virus and variants. The Consensus sequence of the O‐, P‐, Q‐, R‐, and S‐blocks of the NCCR of the archetype is taken from reference (Moens et al. 1995; Moens and Rekvig 2001). The sequences of tissue culture adapted Dunlop strain, Gardner original strain plaque purified strain *wt‐501* and *pm‐522* were used their sequences from the database. It was revealed that BKPyV NCCR derived from *wt‐501* and Vn‐324 had the same structure, and *pm‐522* and Vn1919 had the same structure. The NCCR structure derived from In‐1024 is very similar to *pm‐522*, but the repeated P‐block is unique. The numbers in parentheses indicate the number of bases. Filled triangles indicate a single‐base deletion. The numbers and letters above then indicate base counts from the 5énd of the O‐block and deleted nucleotides.

The NCCR of archetype BK (WW) derived from patient urine is divided into five transcription factor‐binding blocks: O, P, Q, R, and S. We analyzed the NCCR structure of BKPyV derived from three cell lines and compared it with the reported sequences of five blocks (Moens et al. 1995; Moens and Rekvig 2001). The results are shown in Figure 6.

Among the five blocks, the R‐block was absent in all cell lines. Regarding the O‐block, it was approximately the same length in all three cell lines. However, there were minor mutations in Vn‐324 and Vn1919. The NCCR of BKPyV detected in Vn‐324 contained Q‐ and S‐blocks with the same sequence as that of archetype. The BKPyV Gardner strain variant *wt*‐501 also had Q‐ and S‐blocks, same to the BKPyV detected in Vn‐324. Regarding the P‐block, all three cell line‐derived BKPyV or the two virus variants repeat of the P‐block with a partial deletion following the basic 68‐base block. The P‐block of BKPyV derived from Vn‐324 consisted of 69 bases with base addition to the archetype, a second block divided into seven bases and 43 bases parts, and a third block of 68 bases. This three‐time repetition pattern is the same as that of *wt‐501*.

The P‐block of the BKPyV‐derived Vn1919 also had three repeating patterns; however, the positions of the second and third repetition sequences were different from those of Vn‐324. This repeating pattern derived from Vn1919 is exactly the same as that from *pm‐522*. In contrast, the P‐block derived from In‐1024 had the same repetition position as Vn1919 but with two repetitions. In addition, the P blocks from *pm‐522*, Vn1919 and In‐1024 all had a part of the Q block following each repeat (Figure 6).

## 5 Discussion

Recently, many reports have linked to the human polyomavirus BKPyV to human cancer (Polz et al. 2015; Poluschkin et al. 2018; Popik et al. 2019). BKPyV infects over 80% of adults worldwide latently (Rinaldo et al. 2013; Egli et al. 2009; Kean et al. 2009). Therefore, the potential carcinogenicity in humans has been debated. Although a definitive mechanism has not been elucidated, it has been reported to be associated with NCCR mutations in the BKPyV genome (Gosert et al. 2008; Carr et al. 2006; Nukuzuma et al. 2006). BKPyV has an NCCR located between the early and late regions, which regulates bidirectional transcription for viral production and LT‐ag expression. In SV40, which is closely related to BKPyV, LT‐ag stimulates mitosis and binds to the suppressor proteins p53 and RB family proteins, causing the transformation of rodent cells (Li et al. 2003; Lilyestrom et al. 2006). BKPyV LT‐ag, which is highly homologous to the SV40 LT‐ag, may also affect cell proliferation and regulatory mechanisms in neoplastic processes (Harris et al. 1996). Real‐time PCR was used to detect DNA target regions within the BKPyV LT‐ag, may also in three cell lines; Vn‐324, Vn1919, and In‐1024. As reported by Yogo et al. Vn‐324 cells are negative for LT‐ag using immunofluorescence staining. In this study, we confirmed that only Vn‐324 among the three cell lines did not express LT‐ag.

As shown in Figure 3, LT‐ag was detected in the genomic DNA of the three cell lines by PCR, and in the cDNA of Vn1919 and In‐1024 using RT‐PCR. Yogo et al. reported that one copy of the BKPyV genome was integrated into the host genome of Vn‐324 with a breaking point located in the middle of the early region and deletions on both sides of the junction (Yogo et al. 1981). We showed that one copy of BKPyV is present as an episome in Vn1919. However, the PCR product band obtained using Vn‐324 genomic DNA was thinner with lighter than that obtained using Vn1919 genomic DNA. This is thought to be due to the low efficiency of PCR because Vn‐324 is integrated into genomic DNA.

The high intensity of the bands obtained from In‐1024 genomic DNA is thought to be due to the high copy number per genome.

LT‐ag was not detected using immunofluorescence staining in Vn‐324. The reason for the negative immunofluorescence was that LT‐ag was not expressed in Vn‐324 as determined using RT‐PCR. However, the factors responsible for Vn‐324 oncogenesis in the absence of LT‐ag remains unclear. The antigenic epitope used in immunofluorescent staining and the primers and probe used in RT‐PCR are located in close proximity to each other on the genome. It is possible that smaller T antigens that cannot be detected by these methods are expressed. In addition, the possibility that host genome instability or other host transcription factors may be involved in carcinogenesis remains a topic for future research. Another possibility is that mutations in the NCCR may be involved.

The following sections describe the NCCR structure of BKPyV. Archetype NCCRs were sequenced from the viruses isolated from the urine of immunocompromised patients undergoing renal transplantation. BKPyV NCCRs are typical mammalian promoters/enhancers containing many binding motifs for cellular transacting factors in this region. The sequence of the protein‐coding region of the genome is highly conserved, but the NCCR sequence shows considerable variability among BKV isolates due to deletions, duplications, and rearrangements of a basic set of sequence blocks (Moens et al. 1995; Moens and Rekvig 2001). Rearrangement of NCCR has been shown to contribute to host cell tropism, permissiveness, and oncogenicity (Watanabe and Yoshiike 1982; Watanabe et al. 1982; Watanabe and Yoshiike 1986). Three rodent cell lines (In‐1024, Vn‐324, and Vn1919) were established from tumors induced by the experimental injection of the BKPyV mutant into hamsters and mice. The Vn‐324 cell line was established from choroid plexus papilloma, which was induced in hamsters by inoculation with the human polyomavirus BK wild‐type strain *wt*‐501. The Vn1919 cell line was established from ependymomas induced in mice by inoculation with the plaque morphological mutant *pm‐522*. Analysis of the structure of the NCCR sequence in this study showed that the BKPyV NCCR detected in tumor cells showed high homology to the inoculated variants. As shown in Figure 6, the NCCR of BKPyV detected in Vn‐324 has almost the same structure as that of *wt‐501*. Blocks O, P, Q, and S existed, and block P had similar partial repeats. The NCCR of BKPyV detected in Vn1919 had almost the same structure as that of *pm‐522*.

The pattern of partial repeats of block P and the pattern of Q blocks following each repeat were also very similar. Compared with the archetype, *wt‐501* lacked block R, and block P was partially repeated. Additionally, *pm‐522*, which showed stronger tumorigenicity, lacked blocks R and S, had partial repeats of block P, and each repeat was followed by a Q block of different bases. It has been reported that the P block and the boundary with the Q block contain numerous transcription factor binding sequences (Bethge et al. 2015; Moens et al. 1995; Moens and Rekvig 2001).

The Dunlop strain is the best‐characterized laboratory BKPyV strains that grows well in tissue cultured cells. There is a partial repetition in the P‐block, whereas the Q and R blocks are deleted. The repeat pattern of the P block is similar to that of *wt‐501*.

Only the archetype has an R‐block that may not be necessary for in vitro proliferation (Markowitz and Dynan 1988; Moens et al. 1995; Moens and Rekvig 2001). The *wt‐501* strain was isolated from a single stock of Gardner's original strain after several passages in human embryonic kidney cells. The *pm‐522* mutant was rescued from the hamster tumor cell line, Pc‐13, which was established from BKV‐induced hamster pineocytoma. The in vitro transformation ability of this mutant in both hamster and rat cells was higher than that of *wt‐501*. Furthermore, its oncogenicity in hamsters in vivo was also higher (Watanabe et al. 1979; Watanabe and Yoshiike 1982; Watanabe et al. 1982; Watanabe and Yoshiike 1986).

However, it is unclear which variants induced one of the three tumor cell lines, In‐1024.

Regarding BKPyV derived from In‐1024, the NCCR structure differed from both *wt‐501* and *pm‐522*. Only blocks O and P, followed by a partial block Q, were present, and the partial repetition sequence of block P differed from that of *pm‐522*. We discovered that a large deletion (1,184 bp), approximately one‐fourth of the whole viral genome length, existed in BKPyV derived from In‐1024. The deleted areas included the coding regions VP1, VP2, and VP3. Therefore, BKPyV in In‐1024 cells is considered a proliferation defective variant. It is generally believed that these viruses cannot multiply independently in cells on their own (Yoshiike et al. 1982). Human polyomavirus produces numerous defective interfering particles, not only in cultured cells but also in immunocompromised patients (Müller et al. 2018; Henriksen et al. 2014).

During the high‐multiplexity passage of the original Gardner strain in human cells, the replication‐defective variant may have proliferated with the help of a complete variant and dominated by the proliferative capacity acquired by NCCR mutations.

This rearrangement is the result of an evolutionary adaptation to tissue culture cells.

The mechanism underlying NCCR rearrangement as an evolutionary adaptation to new environments remains unclear. However, it is important to note that a latent virus can change its virulence, tumorigenicity, and tropism after reactivation in an immunodeficient state. Further in vitro and in vivo analysis using various mutant strains will be necessary to determine which factors have the greatest influence on the evolution that occurs during virus proliferation.

A novel human polyomavirus, known as Merkel cell polyomavirus, was recently discovered in human Merkel cell carcinoma, a rare and aggressive form of skin cancer that typically develops in older and immunosuppressed individuals (Feng et al. 2008).

These three rodent cell lines serve as important models to future investigate BKPyV‐driven oncogenesis and the evolutionary dynamics of viral NCCR rearrangements.

## Author Contributions

**Setsuko Shioda:** conceptualization, data curation, investigation, methodology, visualization, writing – original draft, writing – review and editing. **Fumio Kasai:** investigation, methodology, data curation, visualization, writing – review and editing. **Midori Ozawa:** investigation, methodology, data curation, visualization. **Azusa Ohtani:** methodology, resources. **Masashi Iemura:** methodology, resources. **Ken Watanabe:** methodology. **Arihiro Kohara:** data curation, funding acquisition, project administration, visualization, writing – review and editing.

## Ethics Statement

The authors have nothing to report.

## Conflicts of Interest

The authors declare no conflicts of interest.

## Supporting information


**Figure 1:** The complete genome of BK polyomavirus strain Gardner 5,190 bp. **Figure 2:** The morphology in the light microscope and immunofluorescence staining LT‐ag of three cell lines. **Figure 3:** Analysis of LT‐ag expression using RT‐PCR. **Figure 4:** Long PCR products with BKV virus check primers. **Figure 5:** BKPyV genome analysis using NGS mapped to the BKPyV Gardner reference strain. **Figure 6:** The NCCR structure of BKPyV derived from three rodent cell lines and the archetype virus and variants. **Table 1:** Primer sequences and location on reference genes, BK polyomavirus strain Gardner. **Table 2:** Copy number of BKPyV DNA sequences per diploid cell, assayed with QuantStudio 3D Digital PCR.

**Supplementary Data 1:** Primers used for primer walking. **Supplementary Data 2:** Amplicon location of NGS sequencing on the reference sequences. **Supplymentary Data 3:** Detection of BKPyV in genomic DNA of three cell lines.

supmat.

## Data Availability

The authors confirm that the data supporting the findings of this study are available within the article and its supplementary materials. Basic, share upon request.
