# Supplementary material for: Human Polyomavirus BK Genome Analysis in BKPyV Induced Rodent Cell Lines
Source: Microbiologyopen. 2025 Sep 11;14(5):e70061. doi: 10.1002/mbo3.70061 (PMC12425812; doi:10.1002/mbo3.70061)
Supplement: Supplementary file 1 — Figure 1: The complete genome of BK polyomavirus strain Gardner 5,190 bp. Figure 2: The morphology in the light microscope and immunofluorescence staining LT‐ag of three cell lines. Figure 3: Analysis of LT‐ag expression using RT‐PCR. Figure 4: Long PCR products with BKV virus check primers. Figure 5: BKPyV genome analysis using NGS mapped to the BKPyV Gardner reference strain. Figure 6: The NCCR structure of BKPyV derived from three rodent cell lines and the archetype virus and variants. Table 1: Primer sequences and location on reference genes, BK polyomavirus strain Gardner. Table 2: Copy number of BKPyV DNA sequences per diploid cell, assayed with QuantStudio 3D Digital PCR. [file MBO3-14-e70061-s003.pptx]

## Slide 1
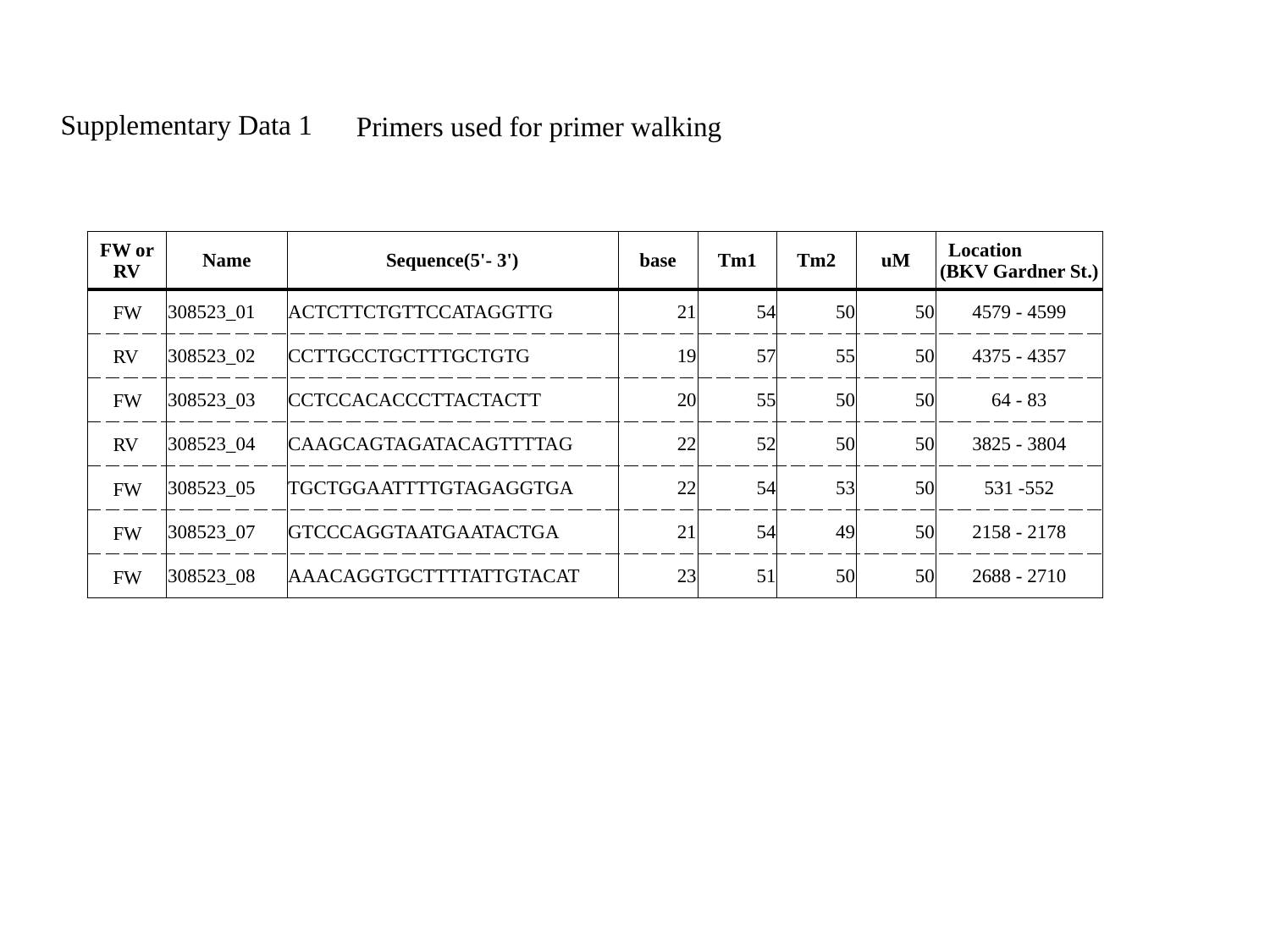

Supplementary Data 1
Primers used for primer walking
| | | | | | | | | |
| --- | --- | --- | --- | --- | --- | --- | --- | --- |
| FW or RV | Name | Sequence(5'- 3') | base | Tm1 | Tm2 | uM | Location (BKV Gardner St.) | |
| FW | 308523\_01 | ACTCTTCTGTTCCATAGGTTG | 21 | 54 | 50 | 50 | 4579 - 4599 | |
| RV | 308523\_02 | CCTTGCCTGCTTTGCTGTG | 19 | 57 | 55 | 50 | 4375 - 4357 | |
| FW | 308523\_03 | CCTCCACACCCTTACTACTT | 20 | 55 | 50 | 50 | 64 - 83 | |
| RV | 308523\_04 | CAAGCAGTAGATACAGTTTTAG | 22 | 52 | 50 | 50 | 3825 - 3804 | |
| FW | 308523\_05 | TGCTGGAATTTTGTAGAGGTGA | 22 | 54 | 53 | 50 | 531 -552 | |
| FW | 308523\_07 | GTCCCAGGTAATGAATACTGA | 21 | 54 | 49 | 50 | 2158 - 2178 | |
| FW | 308523\_08 | AAACAGGTGCTTTTATTGTACAT | 23 | 51 | 50 | 50 | 2688 - 2710 | |

## Slide 2
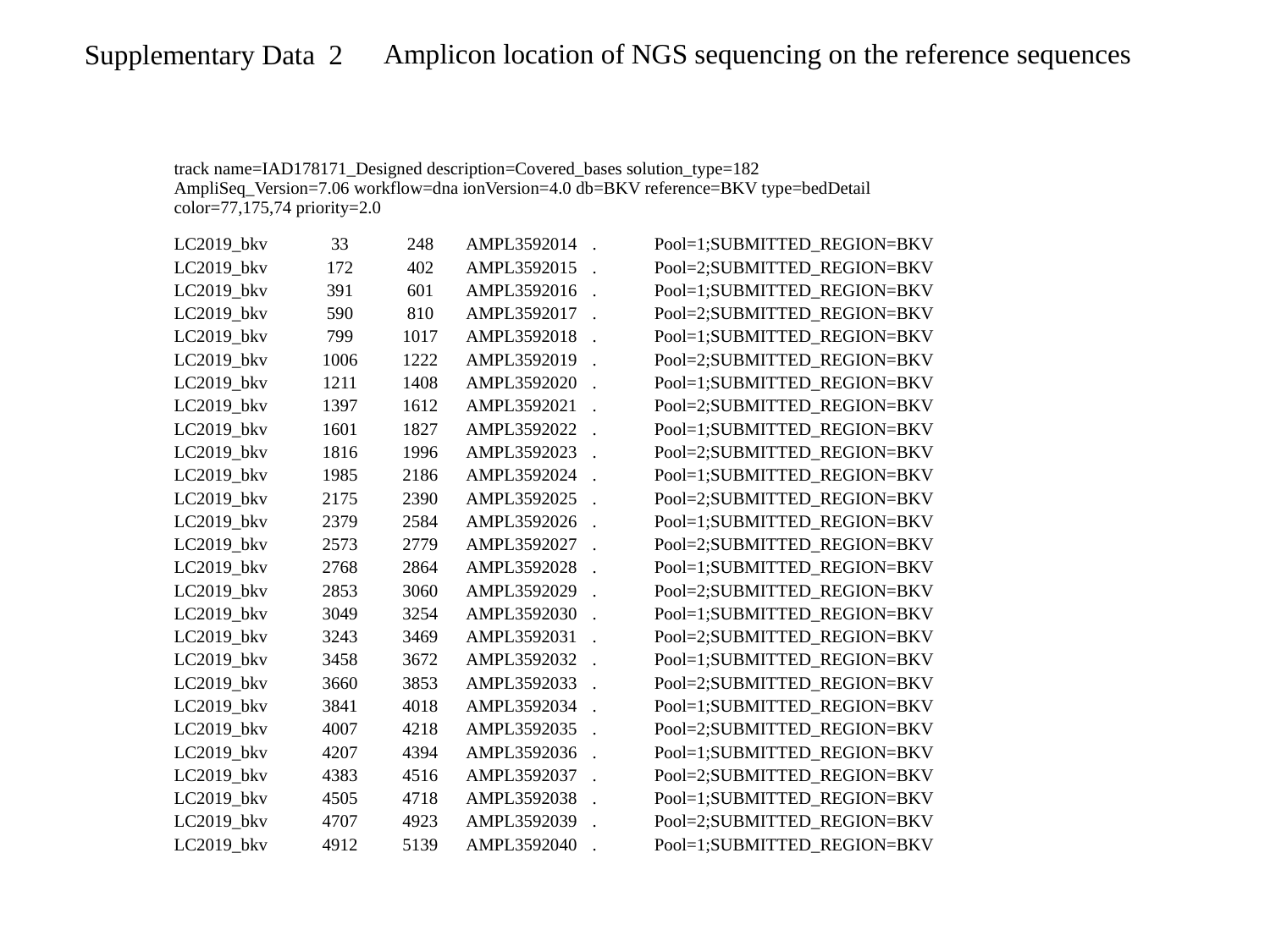

Amplicon location of NGS sequencing on the reference sequences
Supplementary Data 2
| track name=IAD178171\_Designed description=Covered\_bases solution\_type=182 AmpliSeq\_Version=7.06 workflow=dna ionVersion=4.0 db=BKV reference=BKV type=bedDetail color=77,175,74 priority=2.0 | | | | | |
| --- | --- | --- | --- | --- | --- |
| LC2019\_bkv | 33 | 248 | AMPL3592014 | . | Pool=1;SUBMITTED\_REGION=BKV |
| LC2019\_bkv | 172 | 402 | AMPL3592015 | . | Pool=2;SUBMITTED\_REGION=BKV |
| LC2019\_bkv | 391 | 601 | AMPL3592016 | . | Pool=1;SUBMITTED\_REGION=BKV |
| LC2019\_bkv | 590 | 810 | AMPL3592017 | . | Pool=2;SUBMITTED\_REGION=BKV |
| LC2019\_bkv | 799 | 1017 | AMPL3592018 | . | Pool=1;SUBMITTED\_REGION=BKV |
| LC2019\_bkv | 1006 | 1222 | AMPL3592019 | . | Pool=2;SUBMITTED\_REGION=BKV |
| LC2019\_bkv | 1211 | 1408 | AMPL3592020 | . | Pool=1;SUBMITTED\_REGION=BKV |
| LC2019\_bkv | 1397 | 1612 | AMPL3592021 | . | Pool=2;SUBMITTED\_REGION=BKV |
| LC2019\_bkv | 1601 | 1827 | AMPL3592022 | . | Pool=1;SUBMITTED\_REGION=BKV |
| LC2019\_bkv | 1816 | 1996 | AMPL3592023 | . | Pool=2;SUBMITTED\_REGION=BKV |
| LC2019\_bkv | 1985 | 2186 | AMPL3592024 | . | Pool=1;SUBMITTED\_REGION=BKV |
| LC2019\_bkv | 2175 | 2390 | AMPL3592025 | . | Pool=2;SUBMITTED\_REGION=BKV |
| LC2019\_bkv | 2379 | 2584 | AMPL3592026 | . | Pool=1;SUBMITTED\_REGION=BKV |
| LC2019\_bkv | 2573 | 2779 | AMPL3592027 | . | Pool=2;SUBMITTED\_REGION=BKV |
| LC2019\_bkv | 2768 | 2864 | AMPL3592028 | . | Pool=1;SUBMITTED\_REGION=BKV |
| LC2019\_bkv | 2853 | 3060 | AMPL3592029 | . | Pool=2;SUBMITTED\_REGION=BKV |
| LC2019\_bkv | 3049 | 3254 | AMPL3592030 | . | Pool=1;SUBMITTED\_REGION=BKV |
| LC2019\_bkv | 3243 | 3469 | AMPL3592031 | . | Pool=2;SUBMITTED\_REGION=BKV |
| LC2019\_bkv | 3458 | 3672 | AMPL3592032 | . | Pool=1;SUBMITTED\_REGION=BKV |
| LC2019\_bkv | 3660 | 3853 | AMPL3592033 | . | Pool=2;SUBMITTED\_REGION=BKV |
| LC2019\_bkv | 3841 | 4018 | AMPL3592034 | . | Pool=1;SUBMITTED\_REGION=BKV |
| LC2019\_bkv | 4007 | 4218 | AMPL3592035 | . | Pool=2;SUBMITTED\_REGION=BKV |
| LC2019\_bkv | 4207 | 4394 | AMPL3592036 | . | Pool=1;SUBMITTED\_REGION=BKV |
| LC2019\_bkv | 4383 | 4516 | AMPL3592037 | . | Pool=2;SUBMITTED\_REGION=BKV |
| LC2019\_bkv | 4505 | 4718 | AMPL3592038 | . | Pool=1;SUBMITTED\_REGION=BKV |
| LC2019\_bkv | 4707 | 4923 | AMPL3592039 | . | Pool=2;SUBMITTED\_REGION=BKV |
| LC2019\_bkv | 4912 | 5139 | AMPL3592040 | . | Pool=1;SUBMITTED\_REGION=BKV |
