# Supplementary material for: Human Polyomavirus BK Genome Analysis in BKPyV Induced Rodent Cell Lines
Source: Microbiologyopen. 2025 Sep 11;14(5):e70061. doi: 10.1002/mbo3.70061 (PMC12425812; doi:10.1002/mbo3.70061)
Supplement: Supplementary file 3 — supmat. [file MBO3-14-e70061-s001.docx]

Supplementary Data 1. Primers used for primer walking. The sequences of all primers used for primer walking and their locations on the reference genome, BK polyomavirus DNA, complete genome, strain Gardner (Accession: LC029411)

Supplementary Data 2. Amplicon location of NGS sequencing on the reference sequences. The reference sequences are BK polyomavirus DNA, complete genome, strain Gardner (Accession: LC029411). The second and third columns of this table show the reference positions of the amplicons, and the size of each amplicon is approximately 200 bases.

Supplementary Data 3. Detection of BKPyV in genomic DNA of three cell lines BKPyV was detected by real-time PCR (Thermo Fisher Scientific, real-time PCR system 3700), using primers and probe for virus checking. The PCR condition was 45Cycles (95 °C 10▒sec, 60 °C 3▒sec). In the sixth row, the target virus is BKPyV.
